# Correlation between the range of rotation of the hip and the radiographic signs of cam and pincer morphology in femoroacetabular impingement syndrome

**DOI:** 10.1590/0100-3984.2021.0036

**Published:** 2022

**Authors:** Andreza Maroneze da Silva, Flávia Marques Nakatake, Vivian Bertoni Xavier, Vera Lúcia dos Santos Alves, Giancarlo Cavalli Polesello

**Affiliations:** Faculdade de Ciências Médicas da Santa Casa de São Paulo, São Paulo, SP, Brazil.

**Keywords:** Femoroacetabular impingement, Arthroscopy, Hip, Impacto femoroacetabular, Artroscopia, Quadril

## Abstract

**Objective:**

To determine whether hip rotation correlates with the radiographic signs of cam or pincer deformity after hip arthroscopy in patients with femoroacetabular impingement syndrome.

**Materials and Methods:**

This was a single-center retrospective study of data collected between 2014 and 2017. The study sample included 65 patients between 18 and 55 years of age who underwent hip arthroscopy for the treatment of unilateral femoroacetabular impingement. The following data were collected for the periods prior to and six months after surgery range of medial and lateral rotation of the hip; measures on anteroposterior X-rays of the pelvis obtained in the standing position and on ateral X-rays in the Ducroquet profile view; and score on the 33-item International Hip Outcome Tool.

**Results:**

Mean preoperative and postoperative values were as follows: 19.26 ± 10.39° and 30.95 ± 3.52°, respectively, for medial rotation of the hip (*p* < 0.001); 73.85 ± 6.62° and 68.12 ± 5.04°, respectively, for the anteroposterior alpha angle (*p* < 0.001); 56.97 ± 6.09° and 50.61 ± 5.39°, respectively, for the lateral alpha angle (*p* < 0.001); and 0.17 ± 0.11 and 0.07 ± 0.08, respectively, for the acetabular retroversion index (*p* < 0.001). The crossover sign was identified in 75.4% of the patients before surgery and in 44.6% after (*p* < 0.001). Although there was an increase in the range of hip rotation and an improvement in radiographic parameters after arthroscopy, we detected no direct correlation between the two.

**Conclusion:**

Hip arthroscopy can improve medial rotation of the hip, as well as reducing cam and pincer deformities, in patients with femoroacetabular impingement syndrome. However, those findings do not appear to be directly correlated.

## Abstract (pt)

**Objetivo:**

Correlacionar rotação medial do quadril com sinais radiográficos came e *pincer* de pacientes com síndrome do impacto femoroacetabular submetidos a artroscopia.

**Materiais e Métodos:**

Estudo retrospectivo com prontuários consecutivos de 2014 a 2017 em único centro. O estudo incluiu 65 pacientes com impacto femoroacetabular unilateral, de ambos os sexos e idade entre 18 e 55 anos, com indicação de artroscopia do quadril. Os dados colhidos no pré-operatório e pós-operatório de seis meses foram: amplitude de rotação medial e rotação lateral do quadril, mensurações de radiografia anteroposterior da pelve em ortostatismo e perfil de Ducroquet, escala visual analógica de dor e questionário de qualidade de vida International Hip Outcome Tool 33.

**Resultados:**

A amplitude pré-operatória de rotação medial do quadril foi, em média, 19,26 ± 10,39°, e pós-operatória, 30,95 ± 3,52° (*p* < 0,001). Para ângulo alfa anteroposterior, o valor médio pré-operatório foi 73,85 ± 6,62°, e após cirurgia, 68,12 ± 5 , 0 4 ° ( *p* < 0,001). Já o ângulo alfa perfil pré - operatório e pós- operatório foi, respectivamente, 56,97 ± 6,09° e 50,61 ± 5,39° (*p* < 0,001). O sinal do cruzamento foi presente em 75,40% (pré-cirurgia) e após cirurgia em 44,60% (*p* < 0,001). O índice de retroversão acetabular pré-operatório, em média, foi 0,17 ± 0,11, e pós-operatório, 0,07 ± 0,08 (*p* < 0,001). Embora tenham sido observados aumento na amplitude de rotação do quadril e melhora dos parâmetros radiográficos após artroscopia, não houve correlação direta entre as variáveis.

**Conclusão:**

A artroscopia do quadril pode promover normalização da amplitude de rotação medial do quadril e redução dos sinais radiográficos came e *pincer* nos pacientes com síndrome do impacto femoroacetabular, porém, estes achados não possuem correlação direta.

## INTRODUCTION

Femoroacetabular impingement (FAI) syndrome is defined as a change in hip movement characterized by abnormal contact between the femoral head and the anterior aspect of the acetabulum. The symptoms described are pain and limited range of motion, which, together with clinical signs and imaging findings, form the triad of the disease. Its progression correlates strongly with the development of osteoarthritis of the hip(^1^,^2^,^3^).

Limited medial rotation of the hip can generate mechanical overload, as well as changes in strength, neuromuscular control, gait, all of which have a negative impact on the performance of sports activities. In addition, functional activities that require extreme ranges of movement can generate shear force, thus increasing stress on the acetabular labrum and cartilage^(4)^.

Treatment options for FAI syndrome have evolved over the last decade and can be classified as conservative or surgical, producing similar results, although hip arthros-copy is favored in the short term^(5)^. Surgical options include open and arthroscopic resection of bone deformities of the femur or acetabulum, as well as the management of associated soft-tissue lesions (of the labrum or cartilage). The arthroscopic technique is safe, requires less recovery time, and produces consistent results in the short and medium term^(6)^.

Hip arthroscopy for the treatment of FAI has been shown to produce good clinical results in the short and medium term, with gains in range of motion^(7)^. However, no direct correlation has been established between the radiographic parameters of bone morphology and the clinical findings in FAI syndrome. Therefore, the present study was designed with the aim of looking for correlations between the clinical findings of hip rotation and the radiographic parameters, before and after hip arthroscopy in patients with FAI syndrome.

## MATERIALS AND METHODS

This was a retrospective observational clinical study of the medical records of patients evaluated at a private orthopedic clinic. The research project was approved by the local research ethics committee (Reference no. 63881917.8.0000.5479), and the article was prepared in accordance with the Strengthening the Reporting of Observational Studies in Epidemiology guidelines^(8)^.

We analyzed data from consecutive medical records of patients undergoing hip arthroscopy for the treatment of FAI syndrome between January 2014 and April 2017. All procedures were performed by an experienced surgeon, and the rehabilitation was conducted by the same team in all cases. The following inclusion criteria were applied: being between 18 and 55 years of age; having been diagnosed with FAI syndrome; undergoing hip arthroscopy; and undergoing postoperative rehabilitation with a follow-up period of at least six months. Patients who had previously undergone lower-limb orthopedic surgery were excluded, as were those with osteoarthritis, osteonecrosis, or intra-articular hip tumors observed on imaging or arthroscopy, as well as those in whom the radiography protocol was inappropriate for the study and those for whom the clinical data were incomplete.

We identified 160 medical records that were considered eligible. Of those, 95 were excluded, for the following reasons: the patient not meeting the inclusion criteria (n = 35); listing a history of lower-limb orthopedic surgery (n = 5); incomplete data (n = 42); and describing a radiography protocol that was inappropriate for the study (n = 13). Therefore, the study sample comprised 65 complete medical records containing preoperative data and postoperative data for at least the first six months after surgery.

The diagnosis of FAI syndrome was confirmed by clinical examination, radiography, magnetic resonance imaging, and intra-articular injection of anesthetic if necessary(^9^,^10^,^11^). All of the patients underwent an arthroscopic procedure, performed by the same surgeon, who employed a method that is well established in the literature.

Prior to each procedure, the patient was placed in the supine position, on a orthopedic table, with traction applied to both lower limbs. The classic anterolateral and anterior portals were established; an inventory of joint lesions was taken; any pincer deformity was decompressed; and labral debridement or refixation was performed. After the lesions in the central compartment had been treated, the traction was removed in order to access the peripheral compartment. Any cam deformity was removed with an arthroscopic shaver. Before and after arthroscopy, the hip was examined under anesthesia in order to quantify the gain in the range of hip rotation^(12)^.

After hospital discharge, patients were instructed to return at 15 days, three months, six months, and one year, to meet with the surgeon and the rehabilitation team for reassessment and guidance. At each visit, the patients were instructed in how to progress with home exercises to complement the supervised physical therapy sessions at the clinic.

The rehabilitation protocol consisted of five phases^(13)^: maximum protection, in which the objectives were reducing edema, protecting tissue integrity, increasing the smooth range of motion (passive movement and movement on a cycle ergometer), controlling muscle inhibition, and increasing isometric muscle strength; moderate protection, in which the objectives were readjusting the gait, restoring the hip range of motion, restoration of iliopsoas muscle function, and intensifying the strengthening and stabilization of the affected muscles; minimal protection, in which the activities of the previous phases were intensified through deep muscle strengthening exercises, strategies to improve neuromuscular control of the hip, and increases in the volume and intensity of aerobic exercises; functional progression, which was based on specific training in sports and was aimed at achieving further gains in strength, as well as in speed and agility of movement; and sports, in which the patient was allowed to resume sports activities, assuming that there was no pain or muscle compensation and that there was adequate hip range of motion, symmetrical muscle strength, and sufficient scores on functional assessment tests.

Demographic data were collected in order to characterize the sample. Other parameters were analyzed preoperatively and six months after the operation: a pain visual-analogue scale (VAS) score; the score on the 33-item International Hip Outcome Tool (iHOT-33) quality of life questionnaire; the range of medial and lateral rotation of the hip; and the measurements obtained from anteroposterior X-rays of the pelvis obtained in the standing position and from lateral X-rays in the Ducroquet profile view.

The VAS used for pain assessment consisted in patients making a mark on a line numbered from 0 to 10 to indicate the intensity of their pain at the time of the assessments^(14)^. To assess the limitations that FAI syndrome imposes on quality of life, we applied the iHOT-33^(15)^, which has been translated to Portuguese and adapted for use in Brazil^(16)^. The 33 questions on the iHOT-33 are divided into four domains.

Measurements of the range of hip rotation were made by an orthopedic physical therapist with more than five years of experience in the rehabilitation of patients with hip injuries. The measurements were taken with the aid of a goniometer (Carci, São Paulo, Brazil), with the patient in the supine position and the pelvis stabilized with a belt to avoid compensation. The limb evaluated was in hip flexion at 90°, and the contralateral limb was in extension^(17)^. To validate the rotational range of motion measurements, we calculated an intraclass correlation coefficient for two measurements each in 10 hips, with an interval of seven days between measurements, which resulted in values > 0.80.

To identify the radiographic signs and to classify the FAI as the cam, pincer, or mixed type, digital X-rays from the preoperative and (six-month) postoperative periods of arthroscopy were used. The open source freeware Horos, available under the Lesser General Public License, version 3.0, was used. The measurement was performed by an orthopedist with more than five years of experience in hip assessment, using a method that had been validated in a previous study on a related topic^(18)^.

For the impingement to be classified as being caused by a pincer deformity, the anteroposterior X-ray of the pelvis obtained in the standing position should present at least one of the following signs of acetabular retroversion: the crossover sign, characterized by the crossing of the lines of the anterior and posterior acetabular wall within the acetabular cavity; ischial spine sign, characterized by visualization of the ischial spine on an anteroposterior X-ray of the pelvis; the posterior wall sign, characterized by lateralization of the center of the femoral head in relation to the line of the posterior wall of the acetabulum; and an acetabular retroversion index above zero^(19)^. The acetabular retroversion index is calculated by determining the co-efficient between the distance from the lateral acetabular edge to the point where the lines of the acetabular walls cross (crossover point) and the total lateral distance from the acetabular cavity^(20)^:

Acetabular retroversion index=A/[A+B]

where *A* is the distance from the lateral acetabular cavity to the crossing of the lines of the acetabular walls and *B* is the total lateral distance of acetabular cavity.

**Figure 1:**
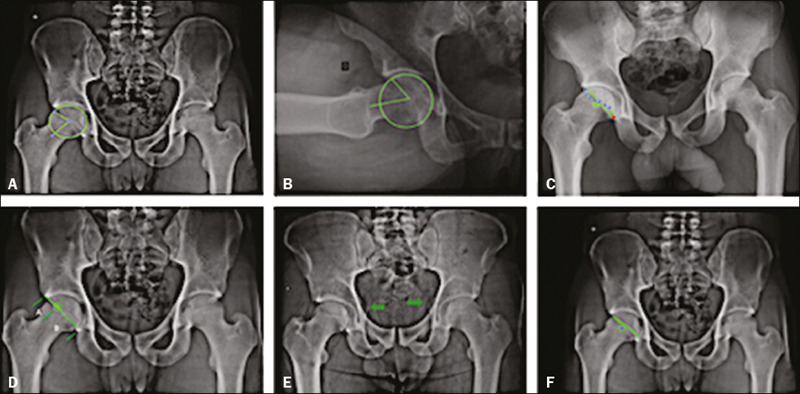
Alpha angle on anteroposterior X-ray (**A**); alpha angle on Ducroquet profile view (**B**); crossover sign (**C**); acetabular retroversion index (**D**); ischial spine sign (**E**); posterior wall sign (**F**).

The impingement was classified as being caused by a cam deformity if the alpha angle was ≥ 69° for men and ≥ 51° for women, as measured on an anteroposterior X-ray obtained in the standing position, or > 55° for either sex, as measured on a lateral X-ray in the Ducroquet profile view. The cases of FAI that were classified as mixed presented a combination of the criteria for the pincer and cam types(^21^,^22^). Figure 1 illustrates all of the measurements and radiographic parameters mentioned above.

Data analysis was performed with Microsoft Excel and with the Statistical Package for the Social Sciences, version 13.0 (SPSS Inc., Chicago, IL, USA). Descriptive data are expressed as mean and standard deviation or as absolute and relative frequencies. Comparisons were made by using the Wilcoxon test, paired Student’s t-test, McNemar’s test, or Pearson’s correlation coefficient, as appropriate. The level of significance was set at *p* ≤ 0.05, and 95% confidence intervals were calculated. Sample calculation was performed for a power of 80%, with an expected correlation coefficient of r = 0.35 and a *p*-value of 0.05. Using the study conducted by Kelly et al.^(7)^ as a reference, we determined that the sample size required was 62 patients.

## RESULTS

The demographic characteristics of the 65 patients in the study sample are presented in Table 1. During the hip arthroscopy, the labrum was reconstructed in one (1.5%) of the 65 cases, whereas it was debrided in three cases (4.6%) and sutured in 61 (93.8%). Resection of cam and pincer deformities was performed in 30 (46.2%) and 58 (89.2%) of the cases, respectively. The cartilage defect was repaired by chondroplasty in one case (1.5%), was repaired by microfracture in three cases (4.6%), and was not addressed in the remaining cases.

**Table:** Preoperative characteristics of the study sample.

Variable	(n = 65)
Gender, n (%)	
Female	39 (60.0)
Male	26 (40.0)
Age (years), mean ± SD	37.66 ± 8.15
Weight (kg), mean ± SD	70.14 ± 13.52
Height (cm), mean ± SD	169.92 ± 9.00
Body mass index (kg/m^2^), mean ± SD	24.12 ± 3.06
Symptomatic limb, n (%)	
Right	40 (61.5)
Left	25 (38.5)
AHA level of physical activity, n (%)	
Sedentary	45 (69.2)
Active	20 (30.8)
Duration of symptoms (months), n (%)	27.35 ± 29.07
VAS pain score (0-10), mean ± SD	5.60 ± 2.50
HOT-33 score (0-100), mean ± SD	41.95 ± 16.62
Location of pain, n (%)	
Inguinal	38 (58.5)
“C” sign	23 (35.4)
Trochanter	4 (6.2)
Type of impingement, n (%)	
Cam	8 (12.3)
*Pincer*	18 (27.7)
Mixed	39 (60.0)

SD, standard deviation; AHA, American Health Association.

There was a statistically significant difference between the preoperative and (six-month) postoperative periods in terms of the medial rotation of the hip, which increased by 11.69° (*p* < 0.001). The data related to the range of motion and the alpha angles are shown in Table 2. The anteroposterior and lateral alpha angles were significantly lower at six months after hip arthroscopy than in the pre-operative period (*p* < 0.001), as were the rate of positivity for the crossover sign and the acetabular retroversion index (*p* < 0.001 for both).

**Table:** Range of motion and radiographic parameters before and after hip arthroscopy (n = 65).

Variável	Preoperative	Postoperative	95% CI	*P*
Medial rotation of the hip at 90°, mean ± SD	19.26 ± 10.39	30.95 ± 3.52	11.69 (9.14–14.23)	< 0.001
Lateral rotation of the hip at 90°, mean ± SD	41.23 ± 6.25	42.65 ± 5.44	1.41 (0.42–2.87)	0.08
Alpha angle on an anteroposterior X-ray, mean ± SD	73.85 ± 6.62	68.12 ± 5.04	5.73 (4.57–6.89)	< 0.001
Alpha angle on a lateral X-ray in the Ducroquet profile view, mean ± SD	56.97 ± 6.09	50.61 ± 5.39	6.35 (4.99–7.72)	< 0.001
Crossover sign, n (%)	49 (75.4)	29 (44.6)	NA	< 0.001
Posterior wall sign, n (%)	32 (49.2)	30 (46.2)	NA	0.625
Ischial spine sign, n (%)	27 (41.5)	27 (41.5)	NA	1.00
Acetabular retroversion index, mean ± SD	0.17 ± 0.11	0.07 ± 0.08	NA	< 0.001

SD, standard deviation; 95% CI, confidence interval at 95%; NA, not applicable.

We observed no statistically significant correlation between the postoperative gain in the range of medial rotation of the hip and the postoperative improvement in radiographic signs. Therefore, it was not possible to establish a relationship between the two, and the Pearson’s correlation coefficient was disregarded. The correlations between the delta values are shown in Table 3.

**Table:** Correlation between the evolution of the range of medial rotation of the hip and that of the radiographic signs of cam and pincer deformities, after arthroscopy (n = 65).

Parameter	Correlation with the Δ range of medial rotation of the hip	
	r	*P*
Δ Anteroposterior alpha angle	0,017	0,892
Δ Lateral (profile) alpha angle	0,165	0,188
Δ Acetabular retroversion index	0,111	0,378

Δ, postoperative vs. preoperative.

**Figure 2:**
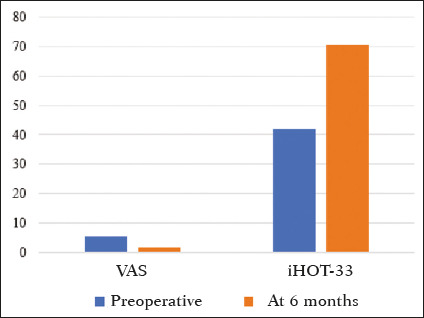
Patient-reported VAS pain scores and iHOT-33 scores, preoperatively and six months after hip arthroscopy.

As can be seen in Figure 2, the mean iHOT-33 score increased significantly, from 41.95 ± 16.62 in the preoperative period to 70.45 ± 16.18 at six months after the surgery (*p* < 0.001). There was also a significant reduction in the mean VAS pain score, which decreased from 5.61 ± 2.53 in the preoperative period to 1.76 ± 2.14 at six months after the surgery (*p* < 0.001).

## DISCUSSION

In our sample of patients undergoing hip arthroscopy, we observed significant differences between the preoperative and (six-month) postoperative periods in terms of the range of medial rotation of the hip, alpha angle values, rate of positivity for the crossover sign, acetabular retroversion index, iHOT-33 score, and VAS pain score. The postoperative gain in the range of medial rotation of the hip was not found to correlate with the presence of a cam or pincer deformity.

There is evidence that diminished range of medial rotation of the hip is a risk factor for the development of intra-articular injury^(2)^. In the present study, the removal of the mechanical block in conjunction with postoperative rehabilitation resulted in an average gain of 11.69° in the medial rotation of the hip at six months after surgery, which is in agreement with the findings of Kelly et al.^(7)^, who followed a cohort of 56 patients undergoing arthroscopy and found a significant mean postoperative gain of 17.70°. In a sample of 109 patients, Choi et al.^(23)^ reported a significant increase in the mean range of medial rotation of the hip at six months after arthroscopic surgery, that range being maintained for up to two years, a result similar to that reported by Stähelin et al.^(24)^.

Prolonged functional activities, which require a greater range of motion than do physiological movements, can generate shear force and contribute to increased stress on the acetabular labrum and cartilage in patients with FAI syndrome^(4)^. The results of the present study demonstrate that arthroscopy can promote gains in the range of medial rotation of the hip and help restore joint mechanics close to the physiological condition, as well as promoting better quality of life in the short term.

Although the patients in our sample did not present extreme alpha angle values, there was a significant reduction in the anteroposterior alpha angle at six months after hip arthroscopy. Some authors have suggested that surgical restoration of the alpha angle to below 55° provides benefits for patients with FAI syndrome^(25)^. One systematic review and meta-analysis that evaluated the outcomes of arthroscopic treatment in 31 studies showed a mean difference in the alpha angle greater than that observed in our study, with evolution and wide variation in radiographic measurements for the analysis of the cam deformity^(26)^.

It may be more difficult to identify and determine the severity of a pincer deformity than a cam deformity because of variability in the measurement, patient positioning, and correlation of the radiographic parameters. In our sample, pincer-type impingement was identified in 27.7% of the patients and mixed-type impingement was identified in 60.0%. In addition to using measures of acetabular retroversion to classify the impingement and assess postoperative changes, we decided to add the acetabular retroversion index, which quantifies the anterior overcoverage of the femoral head, can be a predictor of intraoperative chondral injury and can be incorporated into the preoperative arthroscopic measurements.

In the present study, most of the patients underwent resection of the acetabular rim for treatment of a pincer deformity, resulting in significant reductions in positivity for the crossover sign and in the acetabular retroversion index. We believe that positivity for the ischial spine sign and the posterior wall sign did not change after the surgical procedure, because spatial reorientation of the acetabular cavity (osteotomy) was not performed. Despite the significant gain in the range of medial rotation of the hip and the reduction in the radiographic signs of cam and pincer deformities, no correlations were found between the two.

In the literature, there is an ongoing search for correlations between surgical and clinical/radiographic parameters in an attempt to establish a coefficient to guide surgical interventions and further research^(27)^. It is believed that osteoplasty is not the only factor that determines the postoperative gain in the range of medial rotation of the hip and that other factors may contribute by acting on pain, capsular contracture, and scarring-related adhesions. Although surgery reduces the presence of radiographic signs of impingement, we believe that, regardless of the angle observed on X-rays, each case requires a specific approach, which will provide a functional range of motion.

Postoperative rehabilitation has been recognized as an integral factor in the clinical outcome of hip arthros-copy^(28)^. In the present study, healing time was respected, weight bearing was controlled, and the therapeutic progression was guided on the basis of validated instruments, all of which may have contributed to the gain in range of motion among the patients in our sample. Passive movements of circumduction and optimization of the range of motion in hip flexion can be key elements for early improvements in movement, as well as reducing the nociceptive input from the affected extra-articular structures during the postoperative rehabilitation.

The few studies that have compared surgical and conservative treatment of FAI syndrome have reported conflicting results in the short term(^5^,^29^). The fact that the present study was observational and retrospective precluded any comparison with a rehabilitation control group, although that will certainly be the focus of future studies.

Short- and medium-term results support the notion that hip arthroscopy for FAI syndrome can reduce pain, improve function, and restore range of motion(^30^,^31^). In the present study, there was a significant difference between the preoperative and postoperative periods in terms of the avarage VAS score. Although the VAS is a quantitative measure for the assessment of acute and chronic pain, it is difficult to interpret changes in pain after arthroscopy. That is why we also chose to use a functional scale that would complement the assessment of patient satisfaction after the surgical procedure.

The assessment performed with the iHOT-33 questionnaire showed the postoperative evolution of patient quality of life. Our findings are in keeping with those of Kierkegaard et al.^(30)^, who analyzed 22 studies in a systematic review. Those authors concluded that from three to six months after arthroscopy, patients show a reduction in pain and a functional improvement in the performance of activities of daily living, and that between six months and one year after arthroscopy, patients resume their sports activities.

Our study has some limitations. Because it was retrospective study of data previously collected in medical records, it was subject to information bias. In addition, the number of patients in our sample was small in comparison with those of recognized international multicenter studies. Furthermore, the follow-up period was too short to confirm whether the observed changes persist over the long term and only one radiographic criterion was applied in order to diagnose pincer-type FAI syndrome, which may have resulted in the overestimation of its prevalence. However, the data were collected consecutively and the sample size was calculated as described in a related work in the literature. The interobserver agreement coefficient was excellent, which minimizes the possibility of a measurement bias, and the diagnostic criteria for classifying the types of FAI syndrome were based on the recent literature. The six-month follow-up period, albeit relatively short, was sufficient for complete healing of soft-tissue injuries and to establish a correlation with radiographic parameters. We believe that resolution of the abnormal bone contact was not the only factor responsible for the gain in the range of medial rotation of the hip and that the chosen follow-up time was sufficient to observe functional improvement in the patients.

Research into FAI syndrome is expanding, and the results of the present study can advance understanding of the short-term clinical and radiographic repercussions of hip arthroscopy. Future directions for this work include longer follow-up periods and the inclusion of clinical measures that more closely approximate the functionality of patients.

## CONCLUSION

Hip arthroscopy can promote normalization of the range of medial rotation of the hip and reductions in the radiographic signs of cam and pincer deformities in patients with FAI syndrome. However, there are no apparent correlations between those improvements.
